# Considerations for applying bioethics norms to a biopharmaceutical industry setting

**DOI:** 10.1186/s12910-021-00600-y

**Published:** 2021-03-25

**Authors:** Luann E. Van Campen, Tatjana Poplazarova, Donald G. Therasse, Michael Turik, The Biopharmaceutical Bioethics Working Group, Ariella Kelman, Angela Rossetti, Curtis Chang, Kathleen Novak Stern, Wendell Fortson

**Affiliations:** 1 Ethics Matters, LLC, 5868 E. 71st Street, E-125, Indianapolis, IN 46220 USA; 2 Eli Lilly and Company, Indianapolis, IN USA; 3 GlaxoSmithKline Vaccines, Wavre, Belgium; 4 Indiana University Institutional Review Board, Indianapolis, IN USA; 5 Genentech, a member of the Roche Group, South San Francisco, CA USA; 6 Albert Einstein College of Medicine, Bronx, NY USA; 7 Takeda Pharmaceutical Company, Boston, MA USA; 8 Takeda Pharmaceutical Company, Bannockburn, IL USA; 9 Factor, Chicago, IL USA

**Keywords:** Bioethics norms, Biopharmaceutical industry, Context, Setting, Specification, Definition

## Abstract

**Background:**

The biopharmaceutical industry operates at the intersection of life sciences, clinical research, clinical care, public health, and business, which presents distinct operational and ethical challenges. This setting merits focused bioethics consideration to complement legal compliance and business ethics efforts. However, bioethics as applied to a biopharmaceutical industry setting often is construed either too broadly or too narrowly with little examination of its proper scope.

**Main text:**

Any institution with a scientific or healthcare mission should engage bioethics norms to navigate ethical issues that arise from the conduct of biomedical research, delivery of clinical care, or implementation of public health programs. It is reasonable to assume that while bioethics norms must remain constant, their application will vary depending on the characteristics of a given setting. Context “specification” substantively refines ethics norms for a particular discipline or setting and is an expected, needed and progressive ethical activity. In order for this activity to be meaningful, the scope for bioethics application and the relevant contextual factors of the setting need to be delineated and appreciated. This paper defines biopharmaceutical bioethics as: *the application of bioethics norms (concepts, principles, and rules) to the research, development, supply, commercialization, and clinical use of biopharmaceutical healthcare products*. It provides commentary on this definition, and presents five contextual factors that need to be considered when applying bioethics norms to a biopharmaceutical industry setting: (1) dual missions; (2) timely and pragmatic guidance; (3) resource stewardship; (4) multiple stakeholders; and (5) operational complexity.

**Conclusion:**

Understanding the scope of the biopharmaceutical enterprise and contextual factors of a biopharmaceutical industry setting is foundational for the application of bioethics norms. Establishing a common language and approach for biopharmaceutical bioethics will facilitate breadth and depth of discussion and subsequent implementation to benefit patients, the healthcare system and society.

## Background

Research-based biopharmaceutical companies deliver medicines and vaccines to patients by means of innovative research that occurs in a dynamic and complex ecosystem. Operating at the intersection of life sciences, clinical research, clinical care, public health, and business presents distinct operational and ethical challenges. Ethical integrity in the research, development, manufacturing, and commercialization processes is foundational to the delivery of safe and effective products and provision of reliable and credible information to support their appropriate use. Therefore, companies have a responsibility to patients and other stakeholders to carry out their activities in accordance with applicable public policy (i.e., laws, statutes, regulations), industry guidelines [1], and ethics standards.

As life sciences research and clinical care become increasingly complex, the biopharmaceutical enterprise frequently faces issues with important ethical implications that may not be fully addressed by public policy or industry guidelines. Current examples include access to medicines, drug shortages; use of human biological samples; genomics; use of “big data”; personalized medicine; quality of life and regenerative medicines; data transparency; research in low resource settings; clinical trial diversity; and most recently, the conduct of clinical trials during a pandemic. Systematic analysis and application of ethics standards provides needed guidance for ‘gray areas’ like these topics that lack specific policy or guidelines.

Bioethics is a field of study that examines ethical issues in life sciences, medicine and healthcare. It provides direction to navigate ethical uncertainty associated with these disciplines, as well as rationale for related public policies [2] and discipline-specific guidelines. Any institution with a scientific or healthcare mission should engage with bioethics—whether it be a hospital, research center, clinic, or biopharmaceutical company. Although bioethics norms must remain constant across settings, it is reasonable to assume their application will vary depending on the characteristics of a given setting.

In their seminal book, *Principles of Biomedical Ethics*, Beauchamp and Childress explain that although ethics norms (concepts, principles and rules) are foundational to a common morality, they lack specificity [3]. ‘Practical’ or ‘applied’ ethics, such as bioethics or business ethics, interpret ethics norms for the purpose of generating action-guiding content specific to a context. However, even within a particular applied ethics domain, ethics norms can and should be further specified to provide discipline- or setting-specific guidance. Bioethics ‘specialties’ such as research ethics, clinical ethics and public health ethics are examples of progressive specification [3].

The concept of specification is evident with the bioethics principle of “respect for persons” or “autonomy”, from which the procedural requirement of informed consent was derived [4]. Although this principle is generalizable, without specification it lacks practicality. To be more pragmatic, informed consent requirements have been specified for different settings. Clinical informed consent focuses on the risks and benefits of individual patient treatment. Clinical research informed consent focuses on an individual’s well-being within the setting of a clinical trial and its associated risks and benefits. Public health informed consent focuses on an individual’s well-being in the context of a societal intervention and its associated risks and benefits.

As Beauchamp and Childress state, specification “does not merely analyze meaning; it adds content” [3] (p. 17). In order for this additional content to be meaningful, it is important to understand the scope and context to which bioethics norms are to be applied. Bioethics as applied to a biopharmaceutical industry setting often is construed either too broadly (merged into a general discussion of ethics) or too narrowly (limited to research ethics) with little examination of its proper scope. Given the influence of the biopharmaceutical industry and the ongoing scrutiny of its ethics, it is necessary to consider how bioethics as a discipline (distinct from but complementary to legal compliance and business ethics) relates to this influential sector of the global healthcare system. As MacDonald states, “It is highly unlikely that an adequate understanding of complex issues in health policy will be possible in the absence of an adequate understanding of one of the major players” [5] (p. W38).

There are scores of articles and a number of books [6–11] that examine and discuss industry ‘ethics’ broadly (including a mix of business ethics, corporate responsibility, legal compliance, and bioethics), and there is substantive bioethics literature on topics that relate to industry activities (for example, those listed in the introduction). However, there are a limited number of articles [12–18] and one book [19] that have explored how and why bioethics as a discipline is being applied within a biopharmaceutical industry context. Even with these efforts, none has defined the scope of biopharmaceutical bioethics nor described bioethically relevant characteristics of an industry setting.

Using Beauchamp and Childress’ perspective on ethics specification as a springboard, this paper presents a definition and scope for *biopharmaceutical bioethics*, and describes five bioethically relevant contextual factors that are generalizable to a biopharmaceutical industry setting. We propose that the scope of the biopharmaceutical enterprise and the distinct contextual factors of a biopharmaceutical industry setting necessitate focused bioethics consideration, and that an understanding of this setting is foundational for application of bioethics norms.

The paper is written specifically through an industry lens because all authors have industry experience, but we acknowledge there are other institutions that also engage in biopharmaceutical research and development (R&D) and commercialization endeavors. These organizations may be subject to many of the same bioethical challenges as the industry and may find the presented concepts relevant. We also acknowledge the industry has obligations to both humans and animals, and that these obligations have unique considerations that deserve their own discussion. This paper addresses human biopharmaceutical bioethics.

## Main text

### Definition and scope of biopharmaceutical bioethics

We define biopharmaceutical bioethics as: “*the application of bioethics norms (concepts, principles, and rules) to the research, development, supply, commercialization, and clinical use of biopharmaceutical healthcare products.*”

Upon first reading, this definition may seem fairly obvious. However, bioethics discussions often focus on one particular aspect of the biopharmaceutical enterprise without considering the whole, so commentary is warranted. The scope of this definition is explained phrase by phrase in the following sections. First, however, “*biopharmaceutical healthcare products*” needs to be clarified. “Biopharmaceutical” can be specific to biologically derived medicinal treatments or vaccines (“biologics”) or can refer to both biologics and traditional chemically derived medicinal treatments [20]. In this paper, “biopharmaceutical” has the broader meaning. “*Healthcare products*” can have broad meaning, but in this paper it refers to medicines, vaccines and diagnostics.

### “*The application of bioethics norms (concepts, principles, and rules)*”

Many company codes of conduct were written for business purposes and were required by the U.S. Sarbanes-Oxley Act of 2002 [21]. Company values and principles play an important direction-setting role for establishing standard operating procedures to protect the interests, rights and well-being of research participants and patients, and protect the integrity of the scientific process [16, 17, 22]. Specific guidance, however, is derived from regulations and from bioethics *concepts* (e.g., character and virtues, values, moral ideals and moral emotions), *principles* (e.g., autonomy, beneficence, nonmaleficence and justice), and *rules* (e.g., informed consent, confidentiality and privacy).

*Application* of bioethics norms should be undertaken at two levels. The first is at a company guidance level, such as a company’s position or policy on a topic like clinical development of biopharmaceutical healthcare products for pediatric use. The second is at a case-specific level, such as a clinical development team’s decision whether to use an outcome-adaptive clinical trial design for a specific pediatric study. Both levels are asking the same fundamental questions: (a) *Whether* it is ethical to do X?; and (b) If ‘yes’, then *how* can X be done ethically? Sometimes the “how” question will determine if the “whether” question can be answered affirmatively. For example, it is considered ethically permissible to conduct clinical research with children (“whether”), provided the research is conducted with special protections (“how”). To answer these two questions at a company guidance level bioethics norms are specified, while at a case-specific level they are balanced. To continue with Beauchamp & Childress (p. 18):

Specification entails a substantive refinement of the range and scope of norms, whereas, balancing consists of deliberation and judgment about the relative weights or strengths of norms. Balancing is especially important for reaching judgments in individual cases, and specification is especially useful for policy development [23].

**Fig. 1:**
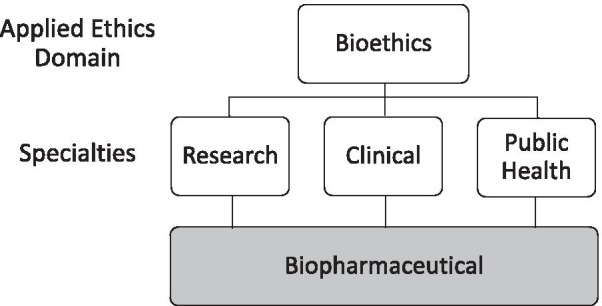
Bioethics specialties. Biopharmaceutical bioethics utilizes ethics norms from the bioethics specialties of research ethics, clinical ethics and public health ethics, and specifies them for the biopharmaceutical industry context

The multidimensional nature of the biopharmaceutical enterprise necessitates application of guidelines from several bioethics specialties—research, clinical and public health ethics (see Fig. 1). This engagement is highlighted throughout the paper. Although not the focus of this paper, the biopharmaceutical enterprise also intersects with business ethics [7, 24–26] and organization ethics [27]. The former addresses general business conduct and the latter addresses corporate culture and processes. Applied ethics domains are not necessarily mutually exclusive and should be integrated when appropriate, including integration with professional codes of conduct (e.g., medicine, nursing, science, etc.). As explained by Drews [25], the obligations derived from medical, scientific or corporate codes of ethics “are not incompatible a priori” (p. 27), but they can conflict with each other in important ways. A physician or scientist in a corporate setting can find it challenging to be bound by all three. Facilitating such integration takes intentional, system-wide effort that acknowledges the challenges.

### “*related to the research, development,*”

‘*Research’* refers to scientific activities that precede or support human clinical trials of a novel investigational biopharmaceutical product or line extension (i.e., new use) for an already-approved healthcare product. This includes basic research, drug discovery and preclinical testing [28]. The focus of research is to identify, evaluate and optimize molecular or biologic entities. Once an entity demonstrates sufficient pharmacological activity and is deemed safe enough to test in humans, then it becomes a ‘candidate’ product and moves to clinical development.

‘*Development’* refers to activities associated with planning and conducting human clinical trials (Phases 1–3) designed to demonstrate safety and efficacy of a new healthcare product or new indication. Development also encompasses activities requisite for submitting an application to regulators and introducing a new healthcare product or new indication into the market [28].

During R&D, research ethics guidance [1, 2, 4, 29, 30] is of obvious importance because it addresses ethical aspects of the design and conduct of research studies; collection, analysis, reporting, publication and sharing of study data; treatment and protection of research participants; and aspects of scientific misconduct. However, R&D activities and directional decisions made at this time can affect not only research participants, but also future patients and prescribing patterns. Therefore, limiting discussions of biopharmaceutical bioethics to research ethics is not sufficient. Engaging guidance from clinical [3, 31] and public health ethics [32, 33] also is important. The former is important because it addresses individual healthcare delivery and the latter because it addresses public health action, which is particularly relevant for infectious diseases vaccines, treatments and diagnostic tests.

### “*supply*, *commercialization, and clinical use of biopharmaceutical healthcare products.”*

Industry activities are more extensive than R&D. They also include supply and commercialization of biopharmaceutical healthcare products. *Commercialization* involves both availability of and accessibility to biopharmaceutical healthcare products. The term ‘availability’ refers to whether a product can reasonably be offered to a specific market, which requires review and approval by local regulators as well as adequate manufacturing capacity and supply. ‘Accessibility’ refers to whether patients, healthcare practitioners and healthcare systems can reasonably acquire biopharmaceutical healthcare products when needed, which is dependent upon adequate supply chains and distribution networks and is influenced by local formulary decisions (considering multiple clinical and economic factors), price, and reimbursement.

Biopharmaceutical companies have a direct responsibility to work towards availability, but cannot control regulatory decisions. Responsibilities for accessibility are shared among manufacturers and healthcare delivery and reimbursement systems, which vary globally. In addition to approved biopharmaceutical healthcare products, access also can relate to investigational products still in development. Access to investigational products is referred to by many names, such as early access, expanded access, “right-to-try”, and compassionate use, among others.

Supply and commercialization activities primarily relate to business ethics [34], but bioethics can help guide decisions that could impact *clinical use* of the product. Biopharmaceutical bioethics draws upon guidance from clinical ethics to address anticipated individual patient needs—such as disease burden, formulation safety and tolerability, medication adherence, when to start/stop/switch treatment, long-term effects, treatment regimens, and diagnostic test interpretations. It also draws upon public health ethics to address anticipated societal healthcare needs—such as disease burden and prevention, pandemic interventions, societal uses or abuses of prescription drugs, drug shortages, and treatment burdens. By engaging with clinical ethics and public health ethics, biopharmaceutical companies can provide accurate and balanced information to healthcare practitioners and patients to enable the appropriate clinical use of healthcare products. This engagement also can facilitate consistency and fairness for multiple stakeholders.

The commercialization phase of a biopharmaceutical healthcare product also involves post-approval activities; including product marketing, safety surveillance, expansion of approved uses (i.e., new indications), and commitments made to regulatory bodies or payers to conduct further studies of safety, efficacy, health outcomes or real-world evidence. All of these activities can have issues that relate to the bioethics specialties of research, clinical or public health ethics.

### *Definition and scope summary*

The application of bioethics norms is necessary at both a company and case-specific level. The multidimensional nature of the biopharmaceutical healthcare product enterprise connects many aspects of contemporary bioethics—including research, clinical and public health ethics. Guidance from each of these specialties is the foundation for specifying and balancing bioethical responsibilities to multiple industry stakeholders. These responsibilities must be considered at both an individual and societal level and extend throughout the product lifecycle—from research to regulatory approval to post-approval activities. In all deliberations, bioethics guidance and company values and principles should be discussed in parallel to ensure consistent institutional guidance.

### The biopharmaceutical industry context

Bioethics provides needed guidance for issues not directly or incompletely addressed by public or private policy. Therefore, it is a vital component of any healthcare organization’s approach to ethics and a necessary complement to compliance efforts. In order to fulfill this role, what we refer to as “contextual factors” must be taken into consideration. Contextual factors are operating requirements and characteristics specific to a healthcare setting. These factors do not change bioethics norms, but rather provide a frame within which the norms should be interpreted and applied. In other words, contextual factors are critical considerations when developing guidance and deliberating cases.

We present five contextual factors for the biopharmaceutical industry: (1) dual missions; (2) timely and pragmatic guidance; (3) resource stewardship; (4) multiple stakeholders; and (5) operational complexity. These factors were identified as generalizable, inter-related and bioethically relevant based upon the shared industry and bioethics experience of the authors and working group members of this paper. The factors routinely present themselves during company position/policy development and case consultations. The purpose of this discussion is to highlight the five factors rather than to fully delineate how one or more of them can or should influence deliberation. In isolation, these factors are not necessarily unique to the biopharmaceutical industry, but when considered as a whole they comprise a distinct context for application of bioethics norms.

#### Dual missions

All business sectors have dual missions. They have a products/services mission (to produce trustworthy deliverables for the benefit of customers), and they have a commercial mission (to make a profit for the benefit of business owners/investors, whether public or private) [35, 36]. Dual missions are symbiotic in that they are mutually beneficial and one would cease to exist without the other. However, the products/services mission is the foundation for existence, and therefore core. Both of these missions fall under the applied ethics domain of business ethics (and legal compliance), but when the products/services mission relates to healthcare, bioethics norms also must be applied to business conduct.

With regard to the biopharmaceutical industry, the core mission of the enterprise is to innovate and deliver new healthcare products for unmet medical needs (spanning the spectrum from quality-of-life to lifesaving, life-extending or disease-preventing products). The pace of scientific, technical and medical innovation is rapid and companies must stay apprised of developments to be relevant and ethical. Sometimes innovation advances can outpace ethical discussions, making a proactive approach to bioethics essential to guide R&D decisions. Two such examples are stem cell research and genomic editing. Biotechnologies used in both innovations have been advancing quickly and have accompanying ethical implications. Anticipating such issues is an important biopharmaceutical bioethics responsibility.

Regarding the industry’s commercial mission, many argue this is an inherent conflict of interest with the industry’s innovative healthcare products mission. The argument presumes that a biopharmaceutical company’s real motive is to make a profit rather than to deliver on its business purpose to improve patients’ lives. A related argument is that a profit motive at least should be tempered—because healthcare products are inherently different from other products due to their consumable nature, and because patients have potential vulnerabilities that reduce their ability to make autonomous decisions (e.g., vulnerabilities due to the disease, treatment, emotions, relationship power differentials, or lack of choice or access) [37, 38]. Alternatively, some have proposed that (a) profit is a virtue because it fuels an enterprise from which many benefit [11]; (b) a commercially competitive biopharmaceutical industry is the best option for developing safe and effective healthcare products [39]; (c) there is no morally relevant distinction between biopharmaceutical companies and businesses that produce other commodities [40]; and (d) profitability is acceptable as long as the societal benefits are deemed substantial enough to balance the monetary gain. Society’s willingness for an industry to make a profit from the misfortune of ill-health has been referred to as “the grand bargain” [41, 42].

Regardless of the arguments, the industry’s commercial mission is a characteristic that must be acknowledged when deliberating bioethics issues. The challenge is how to ethically manage dual responsibilities to make a profit and contribute to society’s common good. The literature is replete with discussion on the topic of balancing these dual missions. This paper does not aim to resolve the issue, nevertheless, we share several pragmatic ways it can be addressed. First, there needs to be a corporate commitment to bioethics. Operationalizing this commitment falls under the applied ethics domain of organization ethics. There are a number of operational models that can be adapted for an industry setting [12, 13, 27, 43–47], depending on available resources. Whichever model is chosen, a concerted educational effort to make bioethics part of daily discussion facilitates the incorporation of norms into business decisions—serving as an important ballast to commercial factors. It is prudent to keep the bioethics effort organizationally separate from R&D or business units to manage internal bias for specific research efforts or product decisions [18]. Soliciting external expert input is another important exercise for navigating challenging issues; although the professional ethics of this practice is debated due to conflicts of interest and potential corporate pressure to tailor advice to commercial desires [5, 48–51]. Some, however, think the legitimate need for advice can be managed with methodology to mitigate these risks [5, 52, 53].

Second, an approach that integrates business ethics and bioethics is warranted, and some biopharmaceutical companies have begun to speak out on the topic of balancing business issues with ethical responsibilities [54]. Third, a balanced perspective must recognize the needs of multiple stakeholders while prioritizing patient well-being [10, 25, 41, 42]. This, however, is “easier said than done”—not just because of varied business stakeholders but also because of varied patient stakeholders (discussed below).

Finally, it is helpful to acknowledge that financial conflict of interest is not unique to the industry. Dual responsibilities and competing interests also are present in other settings, such as academic and healthcare institutions [24, 55]. The industry can look for opportunities to learn from and collaborate with others in the healthcare system on this issue [5]. There is additional value in public–private partnerships in that they take advantage of the strengths of both types of institutions and can serve as a check and support for one another.

#### Timely and pragmatic guidance

The industry’s need for timely and pragmatic guidance is analogous to a clinical care (‘bedside’) need. In both settings there is an expectation for well-timed and effective healthcare interventions to prevent or cure disease, extend life, or alleviate or mitigate symptoms. In both settings, bioethics discussion must be thoughtful and thorough, but theoretical and lengthy discourse on a pressing issue is not tenable. Additionally, in both settings, the outcome of deliberation needs to be sensible, defined and action oriented.

The settings differ in the speed with which decisions impact patient care. Unlike a clinical setting, the patient impact of a biopharmaceutical industry decision generally is delayed because of the inherently long timeline to develop a healthcare product (on average 10 years [28]). Sometimes the impact can be rather immediate, for example with the recall of an approved healthcare product, but this is not the norm. Delayed impact, however, does not negate the necessity of timely and pragmatic decisions. Deciding whether and when to initiate, pause or terminate an endeavor, or to stay or change the course will affect resource utilization, which ultimately impacts patients and multiple stakeholders of the biopharmaceutical enterprise.

Timely and pragmatic guidance is especially relevant during epidemics or pandemics, as in the case of the 2014 Ebola virus disease (EVD) outbreak and the 2019 coronavirus disease (COVID-19) global pandemic. These types of public health crises can place high demands on the biopharmaceutical industry in two ways. First, there is an urgent need for new healthcare products to address the crisis. Second, there is a critical need for continuing development and commercialization of current products to address prevailing medical conditions.

The first demand compels swift development, manufacturing, and distribution of innovative products without sacrificing quality, safety, and ethics [56]. From a bioethics perspective, there is utmost concern for ethical study designs and protocols, as well as equitable distribution of a successful product—especially for marginalized and vulnerable patient populations. The second demand compels skillful management of ongoing product development, manufacturing, and distribution commitments in light of an unanticipated public health crisis. This involves overseeing internal and external resources and relationships. With regard to clinical trials, the US Food and Drug Administration (FDA) states, “Challenges may arise, for example, from self-isolation, site closures, travel limitations, interruptions to the supply chain for the investigational product, or other considerations if site personnel or trial subjects become infected….” [57]. Timely and pragmatic bioethics advice can assist with making difficult prioritization and operational decisions (i.e., resource stewardship decisions).

Because of time constraints and the need for broad cooperation (both within a company and among industry, academic, and government collaborators) during a public health crisis, bioethics expertise should be proactively included in planning, implementation, and after-action reviews. In other words, bioethics advice should be as much a part of public health crisis management as technical, scientific, clinical, regulatory, etc. advice.

#### Resource stewardship

Resources (human, financial, other) for any institution are finite—whether they be modest or considerable. As such, resource stewardship is critical for institutional sustainability and ultimately for promoting the common good in an ethical way. Upholding stewardship or fiduciary responsibilities is a fundamental tenet of business ethics, but it takes on bioethical overtones when resource decisions relate to patient well-being and protection of scientific integrity.

Appropriately managing failure is one aspect of stewardship that is characteristic of the biopharmaceutical industry. R&D and commercial disappointments are commonplace [58–60] because biomedical research and the practice of medicine are complex and results are difficult to predict. Therefore, as resources are allocated, the potential for or the reality of failure needs to be factored into bioethics deliberations. For example, should a medically important trial be initiated when completion feasibility is uncertain due to slow patient enrollment? Or should R&D programmatic efforts be terminated quickly and resources redirected because of a failed trial(s)? Are the medical/scientific/societal needs great enough to consider additional clinical trials despite initially failed attempts (e.g., Alzheimer’s disease treatment research)? Appropriately managing failure encompasses balancing bioethics responsibilities to multiple stakeholders.

#### Multiple stakeholders

As well-stated by Santoro, “Perhaps no business engages the worlds of science, medicine, economics, health, human rights, government, and social welfare as much as the pharmaceutical industry” [42] (p. 1). The industry has an inherently complex network of varied stakeholders—both internal and external to its operations. A possible (but not exhaustive) list includes patients, patient advocates, research participants and collaborators, independent researchers, business partners, vendors, healthcare practitioners, payers, regulators, healthcare authorities, local communities, policy makers, employees, and investors. Because the industry is but one of many members in a modern healthcare system, a company may have limited ability to directly affect an issue involving multiple parties. Nevertheless, it is a key participant and stakeholders have high expectations that it should uphold its responsibilities across its broad scope of influence. Therefore, discernment of industry-specific roles and responsibilities and how to address them is a fundamental part of bioethics deliberation.

**Fig. 2:**
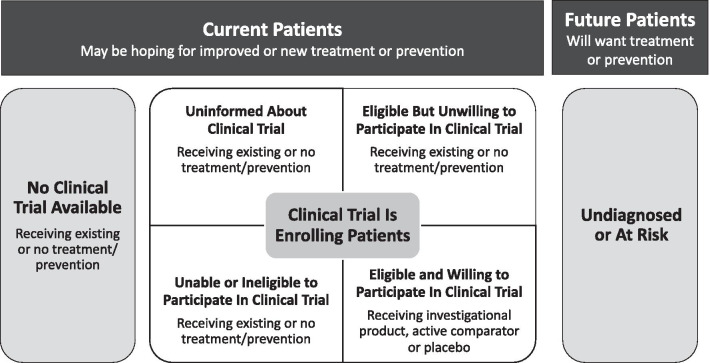
Patient categories. There are two patient categories that must be considered when assessing bioethical responsibilities of the biopharmaceutical industry—current and future patients. Within the category of current patients, there are two subcategories—those for which there is not a clinical trial and those for which there is a clinical trial. Within the latter category, there are four additional subcategories. Most biopharmaceutical bioethics deliberations require consideration of at least two or more categories or subcategories

One of the more challenging aspects of having multiple stakeholders is the variety of patient groups that must be considered. It is appropriate and good to prioritize patient well-being when making decisions involving multiple stakeholders, but often it is not clear which type of patient should receive top priority [22]. Figure 2 depicts a framework to appreciate categories of patients from a clinical trial perspective that may be impacted by a bioethics issue. There can be up to six categories of patients whose medical needs and contributions to research must be considered and prioritized. Identifying patient categories relevant to an issue is an effective way to ensure comprehensive bioethics deliberations. Appreciating the stories of individual patients within these categories is an effective method for avoiding group generalizations [61, 62]. The needs of each relevant group can be evaluated in light of clinical trials factors, such as phase of development, severity of the indication under study, and availability of treatment options.

An illustration of how this framework plays into the assessment of patient-prioritization is that of drug shortages. Drug shortages of a marketed healthcare product present significant challenges as to whether available drug should be diverted to clinical patients or to clinical trial participants. If clinical trial activity is halted or slowed in favor of clinical patients, future patients with a high unmet medical need can suffer. If, however, clinical trial activity is prioritized, current clinical patients with verified medical needs can suffer.

Another illustration is that of early access to investigational biopharmaceutical healthcare products. This topic increasingly is generating bioethical debate in the public and private policy arena, most notably in the United States [63–67]. The debate centers on whether a biopharmaceutical company has an ethical obligation to provide investigational product to patients with a serious or life-threatening disease who have exhausted treatment options. The ethical tension is that company resources are focused on active R&D efforts designed to help future patients with specific indications, but current patients with serious conditions may not be able to wait for a new product approval. Diverting effort away from clinical trials may delay product access for current and future patients waiting for innovative treatment. However, not providing immediate access to an individual could adversely affect the well-being of that patient. The number of stakeholders with interests in this issue is extensive and highlights the need to delineate industry’s role and responsibility(s) to provide treatment outside of clinical trials [68].

Both of these examples underscore an inherent strain between research and public health ethics and clinical ethics. The former two operate in the interest of a patient population and the latter in the interest of one patient. Whether invoking research, public health or clinical ethics, a company has a responsibility to adequately characterize and manage product benefits and risks and give thoughtful consideration to product availability and access for the patient categories represented in Fig. 2.

When issues have multiple stakeholders, it is essential to respect each stakeholder’s values and perspectives on the endeavor(s) to be undertaken. However, it’s important to acknowledge that not all stakeholder views necessarily should have equal weight. As just discussed, patient interests are given greater weight. It’s also important to acknowledge that the likelihood of finding mutually agreeable solutions decreases as the number of stakeholders increases. This can be a difficult reality for decision makers and is why balancing bioethics norms is so important.

#### Operational complexity

To one degree or another operational complexity is a given for the biopharmaceutical enterprise due to global regulatory requirements. The complexity increases for larger companies with an extensive portfolio of products, and international offices and manufacturing facilities. Acknowledging the impact of operational complexity necessitates acknowledging the ripple effect of individual decisions. Bioethics deliberations cannot be conducted in the vacuum of a specific, local situation. Rather, there must be an accounting for broader-scale implications as well as possible future scenarios and outcomes.

*Multilayered structures and simultaneous activities.* A larger operation necessitates multilayered structures to research, develop, manufacture and commercialize new products, and conduct post marketing pharmacovigilance and research. It is common to manage simultaneous preclinical and clinical research efforts in multiple therapeutic areas, which may include codevelopment agreements with other biopharmaceutical entities. For a specific product, research may be conducted at a variety of sites engaging a variety of employees and third parties to assess the safety and efficacy for one or more indications. Additionally, product manufacturing capacity and supply logistics must be developed and maintained, accounting for research needs and anticipated and realized market demand. The multidimensional character of industry activities requires multifactorial consideration during bioethics deliberations.

*Globalization*. A global effort requires comprehensive understanding of large-scale multinational research program logistics and worldwide regulatory, commercialization and distribution requirements. It also requires comprehensive understanding of local health needs, customs, beliefs, expectations, and mores [69]. For example, when deciding whether to conduct clinical trials in resource-poor settings, it is essential to consider how these factors pertain to the appropriateness of the study, volunteer recruitment and incentives, informed consent, and continued access to investigational products. A global effort also necessitates an appreciation of bioethical issues associated with unregulated or inconsistently regulated scientific methodologies. For example, there are no global requirements for gene editing. To be innovative and proactive, companies must discuss the known and anticipated issues of a biotechnology like gene editing [70, 71] as they could affect both public welfare and diverse components of the global biopharmaceutical enterprise (e.g., lab, animal and human research, cell procurement).

### *Contextual factors summary*

Contextual factors are operating requirements and characteristics specific to a given setting. They provide a frame within which bioethics norms are refined and applied. This paper identifies five contextual factors for the biopharmaceutical industry. For a given issue, one or more of them may be in play at either a company guidance or case-specific level.

### Application

There are a number of examples of how bioethics is being applied to the biopharmaceutical industry context. With regard to specification, bioethics position statements and policies are being developed by various companies and posted on their websites. A bioethics framework [16, 17] and a values-based decision-making model [22] have been developed for industry biomedical research. Industry-academic collaborations have generated informed guidance on topics like early access to investigational products [72], clinical trial protocol content [73] and clinical trial conduct in vulnerable populations [74]. With regard to case-specific balancing, in-house consultation services are being incorporated into organizational processes [18], and academic evaluations of industry bioethics decision-making have been conducted [15, 72]. Although these efforts are notable, they have been accomplished with an implicit understanding rather than an explicit articulation of the biopharmaceutical industry context. It is our hope there will be continuing collaborative development of biopharmaceutical bioethics because of the definition and contextual scope and factors presented in this paper.

## Conclusions

Bioethics as applied to a biopharmaceutical industry setting often is construed either too broadly or too narrowly. The purpose of this paper is to articulate its proper scope because understanding the characteristics of a setting is foundational for the application of bioethics norms. In this paper, biopharmaceutical bioethics is defined as: *the application of bioethics norms (concepts, principles, and rules) to the research, development, supply, commercialization, and clinical use of biopharmaceutical healthcare products*. Commentary on the scope of this definition explains that companies have ethical responsibilities to both individuals and society, which extend throughout the product lifecycle. To address these responsibilities, biopharmaceutical bioethics engages with research, clinical and public health ethics. Application of bioethics norms can occur at a company guidance level and at a case-specific level. Deliberations should incorporate both bioethics and company values and principles. Finally, five generalizable, inter-related and bioethically relevant contextual factors need to be acknowledged to frame bioethics deliberations: (1) dual missions; (2) timely and pragmatic guidance; (3) resource stewardship; (4) multiple stakeholders; and (5) operational complexity.

We recognize this characterization will require further refinement. Nevertheless, we conclude that the scope of the biopharmaceutical enterprise and the distinct contextual factors of a biopharmaceutical industry setting necessitate focused bioethics consideration to complement legal compliance and business ethics efforts. The stakes for health and well-being are too high for anything less than substantive discourse on how bioethics can be applied to this influential sector of the healthcare system. Establishing a common language and approach for biopharmaceutical bioethics will facilitate breadth and depth of collaborative discussion and subsequent implementation to benefit patients, the healthcare system and society.

## Data Availability

Not applicable.

## Abbreviations

COVID-19: Coronavirus disease 2019
EVD: Ebola virus disease
R&D: Research and development
US: United States of America
